# Characterization of Unlinked Cases of COVID-19 and Implications for Contact Tracing Measures: Retrospective Analysis of Surveillance Data

**DOI:** 10.2196/30968

**Published:** 2021-11-16

**Authors:** Ka Chun Chong, Katherine Jia, Shui Shan Lee, Chi Tim Hung, Ngai Sze Wong, Francisco Tsz Tsun Lai, Nancy Chau, Carrie Ho Kwan Yam, Tsz Yu Chow, Yuchen Wei, Zihao Guo, Eng Kiong Yeoh

**Affiliations:** 1 Centre for Health Systems and Policy Research Jockey Club School of Public Health and Primary Care The Chinese University of Hong Kong Hong Kong Hong Kong; 2 MRC Centre for Global Infectious Disease Analysis School of Public Health Imperial College London London United Kingdom; 3 Stanley Ho Centre for Emerging Infectious Diseases The Chinese University of Hong Kong Hong Kong Hong Kong; 4 Department of Pharmacology and Pharmacy The University of Hong Kong Hong Kong Hong Kong

**Keywords:** COVID-19, contact tracing, unlinked, superspreading, dispersion, surveillance, monitoring, digital health, testing, transmission, epidemiology, outbreak, spread

## Abstract

**Background:**

Contact tracing and intensive testing programs are essential for controlling the spread of COVID-19. However, conventional contact tracing is resource intensive and may not result in the tracing of all cases due to recall bias and cases not knowing the identity of some close contacts. Few studies have reported the epidemiological features of cases not identified by contact tracing (“unlinked cases”) or described their potential roles in seeding community outbreaks.

**Objective:**

For this study, we characterized the role of unlinked cases in the epidemic by comparing their epidemiological profile with the linked cases; we also estimated their transmission potential across different settings.

**Methods:**

We obtained rapid surveillance data from the government, which contained the line listing of COVID-19 confirmed cases during the first three waves in Hong Kong. We compared the demographics, history of chronic illnesses, epidemiological characteristics, clinical characteristics, and outcomes of linked and unlinked cases. Transmission potentials in different settings were assessed by fitting a negative binomial distribution to the observed offspring distribution.

**Results:**

Time interval from illness onset to hospital admission was longer among unlinked cases than linked cases (median 5.00 days versus 3.78 days; *P*<.001), with a higher proportion of cases whose condition was critical or serious (13.0% versus 8.2%; *P*<.001). The proportion of unlinked cases was associated with an increase in the weekly number of local cases (*P*=.049). Cluster transmissions from the unlinked cases were most frequently identified in household settings, followed by eateries and workplaces, with the estimated probability of cluster transmissions being around 0.4 for households and 0.1-0.3 for the latter two settings.

**Conclusions:**

The unlinked cases were positively associated with time to hospital admission, severity of infection, and epidemic size—implying a need to design and implement digital tracing methods to complement current conventional testing and tracing. To minimize the risk of cluster transmissions from unlinked cases, digital tracing approaches should be effectively applied in high-risk socioeconomic settings, and risk assessments should be conducted to review and adjust the policies.

## Introduction

As COVID-19 cases are still rising around the world and new variants are emerging, nonpharmaceutical interventions (NPIs) are essential for controlling the spread of COVID-19 [1,2] in many countries, especially when vaccination programs are impeded by factors such as vaccine hesitancy, reduced efficacy against some new variants, and vaccine shortage. NPIs such as personal protective equipment, social distancing, contact tracing followed by quarantine, border controls, travel restrictions, and enforced or recommended “stay-at-home” policies and “lockdowns” reduce transmission arising from individual contacts [3].

Hong Kong is a densely populated cosmopolitan city with extensive connections with mainland China and the rest of the world. The city experienced and successfully controlled three waves of COVID-19 in mid-January, March, and July 2020, respectively. Early in the first wave, the government closed its borders with the mainland and enforced a 14-day mandatory quarantine for all arrivals from mainland China and the close contacts of confirmed cases. Quarantine was then extended to arrivals from high-risk countries, and eventually to all arrivals, and entry was denied for all nonresidents during the second wave [1,2]. Unlike the first two waves, during which cases were predominantly imported cases and their close contacts, the third wave was characterized by community transmission in local clusters [3]. In response, the government stepped up public health measures, by implementing mandates for physical distancing, expanded community testing, enhanced case detection, contact tracing, and quarantine [4].

Active identification and isolation of infected persons and quarantine of close contacts reduced subsequent transmissions and brought the third wave under control within two months [3]. The importance and effectiveness of contact tracing have been documented elsewhere [5-8]. According to Aleta et al [5], aggressive social distancing measures, robust testing, contact tracing, and quarantine kept the disease within the capacity of the health care system in the absence of herd immunity.

**Figure 1:**
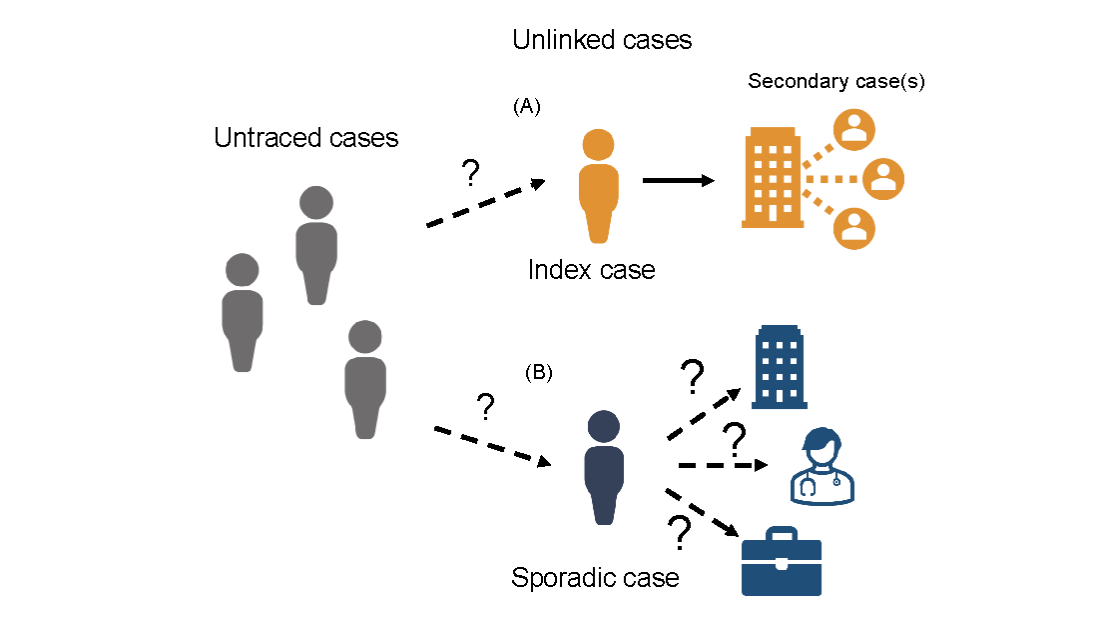
A schematic of the types of unlinked cases in a community. All local cases are supposed to be epidemiologically linked. Untraced cases (in grey) have silent transmissions (dashed arrows), which are the unlinked cases with no information about prior transmission chains. An unlinked case can either be (A) the index case of another cluster or (B) a sporadic case with no identified secondary transmissions. The transmission potential of the unlinked cases can be inferred by the size of the secondary transmission cluster triggered by one case (in orange). For sporadic unlinked cases (in blue), there is no information on whether they caused secondary transmissions or their location history. For each setting u, we estimated the transmission potential of the unlinked cases in that setting by considering a range of probabilities pu (5%-95%) that the sporadic unlinked cases may have visited that setting before.

Ideally, all local cases can be epidemiologically linked, forming a meta-cluster of a closed transmission network in the absence of imported cases. However, in reality, resources for contact tracing are limited, and cases are not able to recollect (ie, recall bias) or choose not to disclose all their close contacts. Close contacts may also not be known to the index case and therefore cannot be traced by conventional methods. As a consequence, some of the cases in a cluster outbreak or in unrecognized chains of transmissions could eventually emerge as “unlinked cases,” either as the index case in a new cluster outbreak or as a sporadic case without known involvement in any identified cluster (Figure 1). As shown by a modelling study [7], unlinked secondary cases can markedly impede outbreak control due to delayed isolation. Another simulation study predicted that at least 71% of close contacts of infectors needed to be traced to control the epidemic (ie, reproduction number reduced to <1) [8].

Hong Kong has implemented stringent containment measures since January 2020 [9,10]. Individuals who contacted a confirmed case up to two days before the initial case’s symptom onset would be put under mandatory quarantine and be tested [11]. Contact tracing became even more important during the third wave, which was characterized by more local transmissions, the impact of which should be evaluated.

It is critical to obtain empirical evidence on the effect of contact tracing on the containment of the epidemic in jurisdictions with control strategies (eg, Singapore, South Korea, and Hong Kong), where intensive contact tracing systems are applied to suppress transmission, to try to avoid the need for extensive restrictions to socioeconomic life (eg, lockdowns). Such data will also be invaluable for informing policies regarding the development of digital tracing technologies that have the potential to minimize the proportion of epidemiologically unlinked cases as well as the perpetuation of outbreaks. In this study, we investigated the clinical and epidemiological characteristics of the unlinked COVID-19 cases in Hong Kong, and estimated the transmission potential of the unlinked cases in different socioeconomic settings. Our study period covers the first three waves of the COVID-19 pandemic in Hong Kong. The findings provide empirical evidence on the value of contact tracing by comparing the epidemiological characteristics of the linked and unlinked cases, and estimating the probability of unlinked cases seeding community outbreaks. We also identified “hot spots” of outbreaks that were triggered by unlinked cases, which can inform risk assessments for designing future public health interventions to control transmission and prevent the health care system from being overwhelmed.

## Methods

### Data Sources

Rapid surveillance data containing the line listing of SARS-CoV-2 infections was obtained from the Centre of Health Protection of the Government of the Hong Kong Special Administrative Region. Individual-level data from January 23 to September 18, 2020, were extracted, including demographics (age, gender, residency), presence of chronic diseases (eg, hypertension, diabetes, and stroke), epidemiological characteristics (imported/local transmission, epidemiological linkages, hospital admission/detection date, mode of detection, types of exposure settings for clusters, any secondary generations linked, and cross-border travel history), clinical characteristics, and outcomes (symptomatic or asymptomatic status, illness onset date, clinical condition, death event). The polymerase chain reaction (PCR) cycle threshold (Ct) value collected on the date closest to the admission date after a 7-day window period was obtained. A lower Ct value suggests a higher viral load.

Asymptomatic cases were those diagnosed without any symptoms of COVID-19 at the time of detection. Modes of detection included the following: (1) medical surveillance, (2) general outpatient clinics (GOPC) or Accident & Emergency (A&E) departments, (3) enhanced surveillance in the private sector, and (4) others (eg, testing for inbound travelers or high-risk groups, and community screening programs). Close contacts of an infected case were quarantined in designated locations and medical surveillance was arranged for other contacts. Time from illness onset to hospital admission was defined as the time interval (days) from illness onset date to hospital admission date.

Ethics approval was obtained from the Joint Chinese University of Hong Kong – New Territories East Cluster Clinical Research Ethics Committee.

### Clusters and Epidemiological Linkages

In accordance with definitions from our previous study, transmission clusters identified by contact tracing may belong to one of the following settings: household, dormitory, workplace, eatery, party, shopping, health care, entertainment, and education. Detailed descriptions of settings can be found in [12]. Household refers to the residential setting where individuals live together most of the time. A dormitory is a room or apartment where a group of unrelated residents reside. A workplace is any working space for and shared by staff. An eatery refers to places where customers stay for meals (eg, cafeterias and restaurants). A party refers to a private social gathering, while a place of entertainment refers to social activities at premises including bars and karaoke bars. A shopping venue can be a market or a department store. A health care setting is where health care services are provided (long-term care facilities are included in this category). In an education setting, teaching and learning activities are carried out—this category includes primary and secondary schools.

### Case Definitions

A linked case is defined as a local secondary case (infectee) epidemiologically linked to a confirmed case (local/imported), either via personal contact or exposure to the same setting at the same time. An unlinked case refers to a local case without any source of infection identified by epidemiological linkage. An unlinked case could be detected either as an index case that has led to further secondary transmission(s) in different socioeconomic settings, or as a sporadic unlinked case without any secondary cases identified (Figure 1).

### Statistical Analysis

As the third wave was predominated by local cases, we divided the data into two epochs: epoch one (from January 23 to June 18, 2020), covering the first and second waves, and epoch two (from June 19 to September 18, 2020), covering the third wave. For each epoch, we compared the categorical variables (ie, age group, sex, symptomatic/asymptomatic, presence of chronic conditions, mode of case detection, ever consulted private clinics prior to admission, critical/serious condition, and death event) of linked and unlinked cases by using chi-square tests, whereas we compared the PCR Ct values of linked and unlinked cases as a continuous variable by using independent *t* tests.

We examined the correlation between the weekly proportion of unlinked cases among all local infections and the weekly number of new local cases using the Spearman correlation coefficient (*r*). For the time interval from illness onset to hospital admission, we compared the survival functions between the linked and unlinked cases on their time to admission using the Kaplan-Meier method. The time to admission was further compared between the two groups when adjusted for age and gender in a Cox proportional hazard model.

The probability that an unlinked case would generate one or more secondary case(s) in a particular setting is estimated by assuming that the offspring distribution (*Z*) follows a negative binomial distribution [13,14]. Compared with other distributions, such as Poisson distribution, the dispersed distributional assumption can account for transmission heterogeneity via specification of the dispersion parameter (*k*) and effective reproductive number (*R*), which have been found to be more rigorous for modeling the offspring distribution when epidemics are characterized by superspreading events [15,16]. Following Lloyd-Smith et al [15], we assumed *Z_u_*, the number of secondary cases generated by a case in a setting *u* (eg, household), follows a negative binomial distribution with mean=*R* and dispersion=*k*. *Z_u_*=0 if a local case did not have any identified secondary cases and thus contributed to a cluster of size *j*=1. For *Z_u_*≥1, *j* would be ≥2 (Figure 1).

By employing a branching process, we formulated the likelihood function (*L_u_*) as follows:




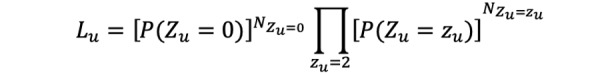




where *N_z_u__=z_u_* is the number of clusters of size *j_u_*. *N_z_u__=z_u_* can only be observed when *j_u_*≥2 (ie, when the case was an index case with traced secondary cases that form a cluster at a specific setting *u*; Figure 1A). Conversely, *N_z_u__=0* is unobserved, as there is no information indicating whether the sporadic unlinked case had been in the particular setting, but only that no further secondary cases were known to be traced or linked to the sporadic case (Figure 1B). Therefore, for each setting *u*, we computed the number of sporadic unlinked cases that have been in or associated with that particular setting by multiplying the total number of sporadic unlinked cases with a percentage *p_u_* (which varied from 5%-95% to account for uncertainty). Since places visited were not mutually exclusive, we assumed *p_u_* to be independent of each other and considered each setting separately. We determined the probability of secondary transmission per unlinked case for each setting *u*, 
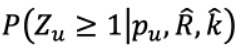
, by solving for the likelihood using the Markov chain Monte Carlo method.

## Results

### Characteristics of Unlinked Cases

There were 438 and 3345 confirmed local cases during epoch one (first and second wave) and epoch two (third wave), respectively. In each epoch, around one-third of the local cases were unlinked cases (ie, not epidemiologically linked with any clusters). Table 1 shows the comparison of the epidemiological characteristics of the linked and unlinked cases. A higher proportion of the unlinked cases were ≥18 years old (*P*<.001) when data from the two epochs were combined. More unlinked cases were male in epoch one (*P*=.02), but this association was not observed in epoch two.

In both epochs, the proportion of asymptomatic cases was lower among the unlinked cases than among the linked cases (9.2% versus 21.2%; *P*<.001). The unlinked cases also had higher Ct values (median 24.8 versus 23.3; *P*<.001), indicating a lower viral load at presentation. Linked cases were more likely to be detected through medical surveillance, whereas unlinked cases were more likely to be detected in GOPCs, A&E departments, and private clinics (*P*<.001). The unlinked cases were also more likely to have consulted private doctors prior to an admission (10.4% versus 4.9%; *P*<.001). For disease outcomes, the mortality rate did not differ significantly between linked and unlinked cases, but unlinked cases were more likely to progress to a critical or serious clinical condition (13.0% versus 8.2%; *P*<.001).

**Table 1:** Comparison of epidemiological characteristics between the linked and unlinked cases in epoch 1 (wave 1 and 2) and epoch 2 (wave 3).

Variables		Epoch 1^a^ (N=438)					Epoch 2^b^ (N=3345)							Overall (N=3783)				
		Linked cases (N=267)	Unlinked cases (N=171)	*P* value^c^		Linked cases (N=2058)		Unlinked cases (N=1287)		*P* value		Linked cases (N=2325)			Unlinked cases (N=1458)		*P* value	
**Age group (in years), n (%)**					.07						<.001							<.001
	<18	12 (4.5)	1 (0.6)			203 (9.9)		24 (1.9)				215 (9.2)			25 (1.7)			
	18-49	161 (60.3)	114 (66.7)			855 (41.5)		552 (42.9)				1016 (43.7)			666 (45.7)			
	50-64	62 (23.2)	41 (24.0)			565 (27.5)		419 (32.6)				627 (27.0)			460 (31.6)			
	≥65	32 (12.0)	15 (8.8)			435 (21.1)		292 (22.7)				467 (20.1)			307 (21.1)			
Male, n (%)		128 (47.9)	101 (59.1)	.02		988 (48.0)		616 (47.9)		.94		1116 (48.0)			717 (49.2)		.48	
Asymptomatic, n (%)		39 (14.6)	9 (5.3)	.002		450 (22.1)		124 (9.7)		<.001		489 (21.2)			133 (9.2)		<.001	
Presence of chronic conditions, n (%)		88 (33.0)	54 (31.6)	.76		241 (11.7)		172 (13.4)		.16		329 (14.2)			226 (15.5)		.25	
**Mode of case detection, n (%)**					<.001						<.001							<.001
	Medical surveillance	145 (54.3)	9 (5.3)			828 (40.2)		65 (5.1)				973 (41.8)			74 (5.1)			
	GOPC^d^ and A&E	66 (24.7)	71 (41.5)			1004 (48.8)		764 (59.4)				1070 (46)			835 (57.3)			
	Private clinics	7 (2.6)	35 (20.5)			196 (9.5)		375 (29.1)				203 (8.7)			410 (28.1)			
	Others^e^	49 (18.4)	56 (32.7)			30 (1.5)		83 (6.4)				79 (3.4)			139 (9.5)			
Ever consulted private clinics prior to admission, n (%)		47 (17.6)	41 (24)	.10		67 (3.3)		111 (8.6)		<.001		114 (4.9)			152 (10.4)		<.001	
PCR Ct^f^ value, median (25th percentile to 75th percentile)		24.4 (19.1-31.1)	29.3 (22.3-33.8)	<.001		23.2 (18.8-28.8)		24.5 (19.8-29.7)		<.001		23.3 (18.8-29.0)			24.8 (19.9-30.1)		<.001	
Critical/serious condition, n (%)		17 (6.4)	17 (9.9)	.17		173 (8.5)		171 (13.4)		<.001		190 (8.2)			188 (13.0)		<.001	
Death, n (%)		3 (1.1)	2 (1.2)	.96		61 (3.0)		34 (2.6)		.59		64 (2.8)			36 (2.5)		.60	

^a^January 23 to June 18, 2020, covering the first and second wave.

^b^June 19 to September 23, 2020, covering the third wave.

^c^*P* values were from chi-square tests and independent two-sample *t* tests for categorical and continuous variables, respectively.

^d^GOPC: general outpatient clinic; A&E: Accident & Emergency department.

^e^Others includes testing for inbound travelers, testing for high-risk groups, and community screening programs.

^f^PCR Ct: polymerase chain reaction cycle threshold.

### Relationship of Linked Cases With Epidemic Size and Time to Hospital Admission

**Figure 2:**
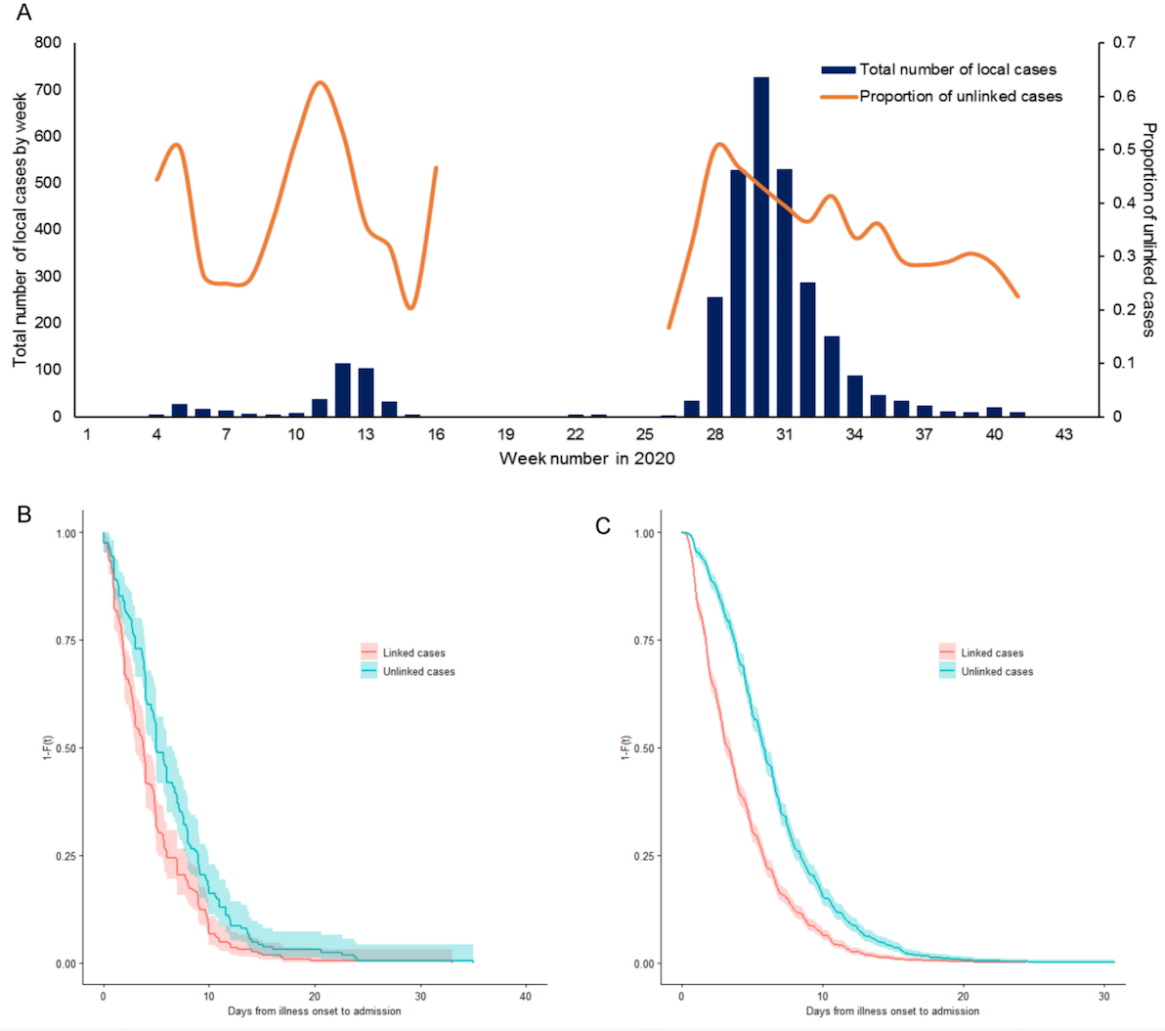
(A) Relationship between the proportion of unlinked cases and weekly number of new local cases (in the week of illness onset). A survival analysis of the time to admission in epoch 1 (B) and epoch 2 (C). The proportion of unlinked cases was smoothed using a 1-week window period.

The proportion of unlinked cases was significantly associated with the number of new local cases by week (*r*=0.39; *P*=.049; Figure 2A). In both epochs, the unlinked cases had significantly longer time to hospital admission than the linked cases (*P*<.001, sex and age adjusted; Figure 2B and Figure 2C). The median time to admission was 3.78 days for the linked cases (95% CI 3.30-4.27) and 5.00 days (95% CI 4.37-5.63) for the unlinked cases in epoch one, and 3.26 days (95% CI 3.06-3.47) and 4.46 days (95% CI 4.28-4.64) in epoch two, respectively.

### Transmission Potential of Unlinked Cases in Different Settings

Of the unlinked cases, 678 (46.5%) were identified as index cases with further secondary transmission. Settings where these cluster transmissions were most likely to be observed were households (493/678, 72.7%), eateries (54/678, 8.0%), and workplaces (48/678, 7.1%).

**Figure 3:**
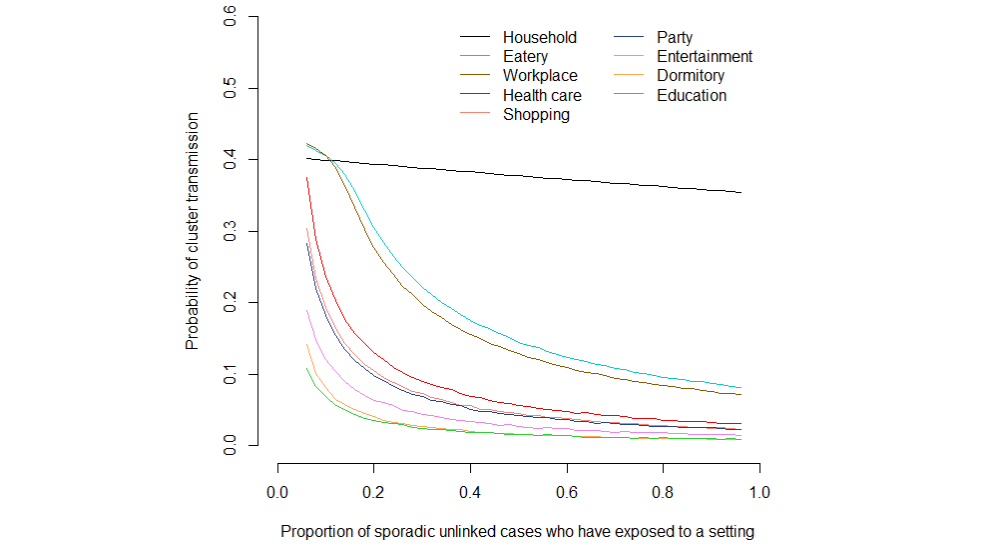
Probabilities of cluster transmissions in different settings across the proportions of unlinked sporadic cases assumed to have visited each setting. The probability of cluster transmissions is defined as the probability of having caused a cluster size >1 for each unlinked case, based on the offspring distribution that follows a negative binomial distribution given the estimated dispersion parameter and effective reproductive number. Since there is no location history for the sporadic unlinked cases, the number of these cases at a particular setting u was simulated by multiplying the total number of sporadic unlinked cases with a factor pu that varied from 5%-95%.

Households had the highest probability of outbreaks—each unlinked case had around 0.4 probability of causing a cluster of size *j*≥2 in household settings (Figure 3). In general, outbreak potential was sensitive to the values of *p_u_* in all settings except households, where the probability of secondary transmission was insensitive to *p_u_*, consistent with a previous study showing a comparatively low potential for dissemination from a household to other settings [12]. Eateries and workplaces also had high outbreak potentials compared to other settings, with probabilities ranging from around 0.1-0.4 under different values of *p_u_*. Health care, shopping, and party settings were particularly sensitive to *p_u_* and could have a secondary transmission probability of >0.3 if a small proportion of sporadic unlinked cases had been involved in these settings (ie, low *p_u_*). An outbreak potential probability as high as 0.3 (or any threshold defining a “high” probability) informs decisions regarding the public health measures needed to mitigate the risk of transmission in those settings.

## Discussion

In this study, we characterized the epidemiological profile of local unlinked cases in the first three epidemic waves of COVID-19 in Hong Kong, a Special Administrative Region with a well-established case detection and contact tracing system used as a key containment strategy. Nevertheless, conventional contact tracing is unable to identify all close contacts at risk of infection, resulting in a significant number of unlinked cases, detected either as sporadic cases or as index cases of clusters of infections. We showed that unlinked cases were positively correlated with time to hospital admission, serious/critical clinical condition, and epidemic size. The higher proportion of patients progressing to a serious/critical condition among the unlinked cases was particularly pronounced in the third wave when cases were mostly due to local transmission.

Compared with the linked cases that were quickly identified through contact tracing and could be diagnosed before symptom onset, unlinked cases—for which no information regarding epidemiological linkage had been obtained through conventional contact tracing methods—could only be ascertained when they sought medical consultation upon becoming symptomatic. This could result in a longer delay in receiving appropriate care and treatment, which could result in an increased likelihood of progressing to a serious/critical clinical condition, which is consistent with reports regarding other respiratory infectious diseases [17,18]. However, the overall mortality rate was not found to be significantly different between linked and unlinked cases. One possible reason is that the health care system provides effective clinical management of patients with COVID-19. In Hong Kong, the mortality rate has been relatively low [19] and the health care system still had the capacity to provide optimal care for patients with COVID-19 as the epidemic was brought under control rapidly with physical distancing measures during both epochs of the epidemic.

Another important finding was the association between the unlinked infections and the local epidemic situation. As indicated by Kretzschmar et al [6], the delay in detection and isolation would reduce the effectiveness of contact tracing measures for COVID-19 control. Across the different settings, the probability of an outbreak was highest in households, a phenomenon reported by studies in mainland China [20] and Korea [21]. However, it should be noted that household members are usually less subject to recall bias than others, leading to an overrepresentation of household secondary cases in our data set and an upward bias in the transmission potential for household settings. As shown by Adam et al [13], frequencies of secondary transmission were highest in households, while clusters were largest in social settings like restaurants, where superspreading events occurred. In line with our finding, a previous study showed that eateries and workplaces were the settings more likely to generate transmission cascades across more than a single setting in the second wave of the epidemic [12]. Such enclosed settings where large numbers of people were in close contact were a risk factor for transmission, while the congregation of people not known to each other was a risk factor for not being able to identify close contacts, allowing the dispersion of infections in a wide range of the settings.

Globally, it was estimated that 10% of cases outside China had caused 80% of secondary cases at the start of the pandemic [22]. Superspreading events have driven the pandemic significantly and could quickly overwhelm the contact tracing system; for example, for a single large cluster, the epidemic investigation team traced up to 1000 people in Hong Kong [23]. Although venues like gymnasiums and sport venues were closed in Hong Kong and the government encouraged people to work from home during the pandemic, most businesses were operational. Dining in in restaurants was permitted with restrictions in numbers, operational hours, and capacity. Unsurprisingly, we found a higher probability of cluster transmissions in eateries and workplaces compared to other settings. However, considering the scenario in which sporadic unlinked cases are not likely to have been involved in clusters in health care, shopping, and party settings, these settings would also have high potentials for cluster transmission. Our analysis thus provides a retrospective risk assessment for optimizing the relaxation of social/physical distancing restrictions in different settings; in these settings, risk can be managed with a robust and strictly enforced digital tracing system. Resurgences of infections could be prevented, obviating the need for a complete lockdown and the associated social and economic costs.

Many studies have focused on identifying hot spots for superspreading events. Although indoor environments with poor ventilation are generally riskier, human activities and behaviors (whether people have taken precautions) are even more crucial [12,24]. Therefore, health care, shopping, and party settings should have strictly implemented precautions for preventing cluster transmission, because of the risk and large number of contacts associated with such settings. Our findings offer empirical evidence for the differential influence of unlinked cases on epidemic control, which could help prioritize mitigation strategies.

Many jurisdictions in the Western Pacific Region heavily relied on stringent case detection, contact tracing, isolation, quarantine, and intensive testing programs together with border restrictions for COVID-19 control [19]. However, the conventional contact tracing approach is labor-intensive and feasible for low-incidence settings only [7], capturing only known contacts and being subject to cases’ recall bias. Mobile phone data are a promising complementary tool. First, mobility data can inform incidence forecasts—for example, modeling combined with local mobility data showed that restaurants, bars, and gyms were hot spots for transmission [25]. Second, apps can be used to alert users instantaneously if their contacts are confirmed positive and recorded in the system [26,27]. This would prompt the alerted users to seek testing, especially for presymptomatic or asymptomatic transmissions [28-31]. In this study, we found that the percentage of asymptomatic infections was significantly lower in the unlinked cases than in the linked cases, primarily due to the fact that the unlinked cases would only be identified when they became symptomatic and sought medical consultation. However, even though contact tracing can identify some asymptomatic cases, others are missed by the system. App-based tools could prove useful for tracing these infections by providing improved coverage and timeliness and have the potential to address the challenge of anonymous close contacts that the index patients do not know. Digital contact tracing has been deployed in many countries [32], including India [33], Switzerland [26], and the United Kingdom [27]. Nevertheless, we acknowledge that the effectiveness of app-based monitoring tools on outbreak control remains controversial when variations in exposure relative to the infectiveness period, testing accuracy, isolation adherence, and coverage of the app-based technology are taken into account [34,35]. Most importantly, the use of smartphone data for digital contact tracing in such contexts raises a number of ethical and privacy concerns [36]. In Hong Kong, for example, residents were skeptical of the LeaveHomeSafe app implemented after the third wave of the epidemic, despite the government’s reassurance that user registration was not required and the check-in data would not be uploaded to the government’s system or any other systems [37,38]. The critical role of digital technology in complementing conventional contact tracing needs to be evaluated, and ethical and privacy concerns must be addressed.

One major limitation of this study is that the location history for the sporadic unlinked cases was unavailable. We estimated the transmission probability of the unlinked cases by considering a range of scenarios that varied by the proportion of sporadic unlinked cases. In addition, we did not quantify the frequency of outbreaks (eg, the number of clusters per 1000 unlinked cases), which would be of interest for decision-making due to the unknown number of sporadic unlinked cases in each setting. Instead, we provided the probability that an unlinked case could result in secondary transmission to convey the outbreak potential in that setting. This indicates a need for an exposure tracking system that automatically computes the risk level for different settings. Second, cases were linked epidemiologically without validation through other approaches such as the routine use of phylogenetic analysis due to a lack of viral sequencing data [39]. Fingerprinting of SARS-CoV-2 via phylogenetic information can assist in the identification of chains of transmission; therefore, such analyses should be performed in future investigations. Third, the retrospective data cannot infer transmission potential for some settings that were already closed during the study period (eg, kindergartens that were closed since the early phase of the first wave). Fourth, some of the unlinked cases may have been infected by the imported cases exempted from quarantine and testing [40], limiting the generalizability of our results to other places with different border control measures.

In conclusion, our findings suggest a need to promote the use of digital tracing methods on top of current conventional testing and tracing. Contact tracing measures can potentially shorten the time to admission, reduce serious cases, and prevent further spread. With the considerable probability of secondary transmission from the unlinked cases, digital tracing measures should be strictly enforced in high-risk social settings and the local epidemic should be closely monitored such that the measures can be adjusted in a timely manner.

## Abbreviations

A&E: Accident & Emergency
Ct: cycle threshold
GOPC: general outpatient clinic
NPI: nonpharmaceutical intervention
PCR: polymerase chain reaction
